# The genome sequence of the Hawthorn Fruit Fly, *Anomoia purmunda *(Harris, 1780)

**DOI:** 10.12688/wellcomeopenres.22849.1

**Published:** 2024-08-12

**Authors:** Liam M. Crowley, James McCulloch, Nathan Medd, University of Oxford and Wytham Woods Genome Acquisition Lab, Darwin Tree of Life Barcoding collective, Wellcome Sanger Institute Tree of Life Management, Samples and Laboratory team, Wellcome Sanger Institute Scientific Operations: Sequencing Operations, Wellcome Sanger Institute Tree of Life Core Informatics team, Tree of Life Core Informatics collective, Darwin Tree of Life Consortium

**Affiliations:** 1 University of Oxford, Oxford, England, UK; 2 University of Edinburgh, Edinburgh, Scotland, UK

**Keywords:** Anomoia purmunda, Hawthorn Fruit Fly, genome sequence, chromosomal, Diptera

## Abstract

We present a genome assembly from a female Hawthorn Fruit Fly, *Anomoia purmunda* (Arthropoda; Insecta; Diptera; Tephritidae). The genome sequence has a length of 798.30 megabases. Most of the assembly is scaffolded into 6 chromosomal pseudomolecules, including the X sex chromosome. The mitochondrial genome has also been assembled and is 16.48 kilobases in length.

## Species taxonomy

Eukaryota; Opisthokonta; Metazoa; Eumetazoa; Bilateria; Protostomia; Ecdysozoa; Panarthropoda; Arthropoda; Mandibulata; Pancrustacea; Hexapoda; Insecta; Dicondylia; Pterygota; Neoptera; Endopterygota; Diptera; Brachycera; Muscomorpha; Eremoneura; Cyclorrhapha; Schizophora; Acalyptratae; Tephritoidea; Tephritidae; Trypetinae; Trypetini; Chetostomatina; *Anomoia*; *Anomoia purmunda* (Harris, 1780) (NCBI:txid103414).

## Background

*Anomoia purmunda* Harris 1780, also known in the UK as the Hawthorn Fruit Fly or Spectacled Berry Fly, is a ‘picture-winged’ brachyceran Diptera in the family Tephritidae. The body size of the adult ranges from 4 and 6 mm. It exhibits striking wing patterning, a feature distinct enough to allow reliable species-level identification ( White, 1988).

One of the most striking features of adult *A. purmunda* are their intricate wing patterns. The vast majority of tephritid fruit flies possess patterned wings suggestive of visual signalling, either sexual, agonistic, or defensive in nature. In other tephritids with similar patterns it has been suggested that wings may be used in a form of defensive mimicry: the lined pattern resembling the silhouette of co-ocurring predatory jumping spiders (Salticidae) even mimicking the territorial display of said arachnids in movement ( Greene *et al.*, 1987). It is currently unclear whether the wings of *A. purmunda* have such adaptive uses as no observations, either wild or experimental, have been made in this species, a potential area for future behavioural work. 

*Anomoia purmunda* is widely distributed across the temperate Palaearctic ( Kandybina, 1972), and has been detected across much of Europe ( Hoffmeister, 1992; Korneyev & Korneyev, 2015; Smith, 1995; Stalažs, 2014a; Stalažs, 2014b; Tartanus *et al.*, 2021; Teodoru *et al.*, 2015; Thompson & Blunt, 2018; Tuba, 2009) with additional records from South Korea ( GBIF.org, 2024). Its primary plant host is thought to be hawthorn ( *Crataegus* spp *.*) but is associated with a range of other rosaceous shrub genera, including *Berberis, Cotoneaster, Pyracantha, Sorbus, and Hippophae* ( NBN Trust Partnership, 2024; White, 1988). Close associations with host plants are common in this family, and *A. purmunda* may be found in habitats rich in these host plants. This species seems to be attracted to lure traps designed for the important pests in this family: Medfly ( *Ceratitis capitata*) and cherry fruit fly ( *Rhagoletis cerasi*) ( Tartanus *et al.*, 2021).

*A. purmunda* appears to be univoltine with records of adults suggesting a flight period of between June and September in the UK ( NBN Trust Partnership, 2024). Adult females deposit eggs generally under the skin of host fruits which develop through three larval instars, feeding on plant material. Larval development in fruit feeding Tephritidae such as *A. purmunda* take between 15-30 days before pupation in the soil ( White, 1988). Kandybina (1977) noted parasitism by the Pteromalid wasp, *Halticoptera laevigata*.

Here we present a chromosomally complete genome sequence for *Anomoia purmunda*, based on a female specimen from Wytham Woods, Oxfordshire, UK

## Genome sequence report

**Figure 1.**
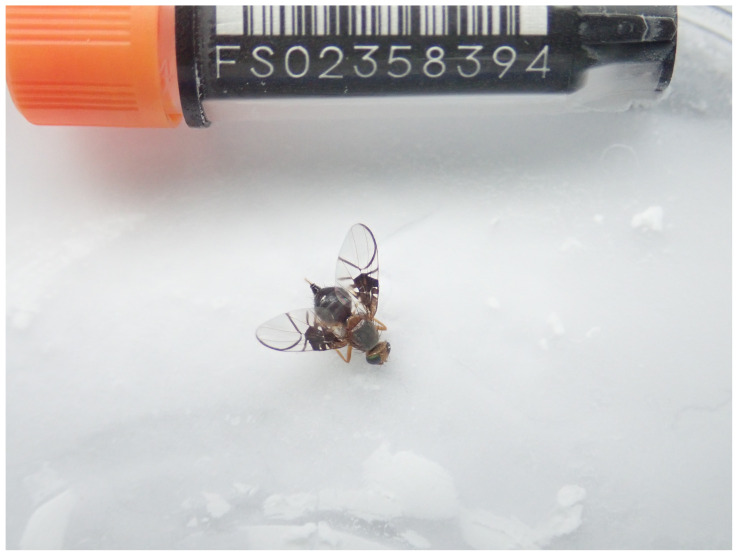
Photograph of the *Anomoia purmunda* (idAnoPurm1) specimen used for genome sequencing.

The genome of an adult female *Anomoia purmunda* ( Figure 1) was sequenced using Pacific Biosciences single-molecule HiFi long reads, generating a total of 25.33 Gb (gigabases) from 2.02 million reads, providing approximately 35-fold coverage. Primary assembly contigs were scaffolded with chromosome conformation Hi-C data, which produced 145.47 Gbp from 963.35 million reads, yielding an approximate coverage of 182-fold. Specimen and sequencing information is summarised in Table 1.

**Table 1.** Specimen and sequencing data for *Anomoia purmunda*.

Project information			
**Study title**	Anomoia purmunda (hawthorn fruit fly)		
**Umbrella BioProject**	PRJEB55940		
**Species**	*Anomoia purmunda*		
**BioSample**	SAMEA7520325		
**NCBI taxonomy ID**	103414		
Specimen information			
**Technology**	**ToLID**	**BioSample accession**	**Organism part**
**PacBio long read sequencing**	idAnoPurm1	SAMEA7520397	Whole organism
**Hi-C sequencing**	idAnoPurm2	SAMEA112232991	Whole organism
**RNA sequencing**	idAnoPurm3	SAMEA112233354	Whole organism
Sequencing information			
**Platform**	**Run accession**	**Read count**	**Base count (Gb)**
**Hi-C Illumina NovaSeq 6000**	ERR11242505	9.63e+08	145.47
**PacBio Sequel II**	ERR11359861	2.02e+06	25.33
**RNA Illumina NovaSeq 6000**	ERR11837454	6.51e+07	9.83

**Figure 2.**
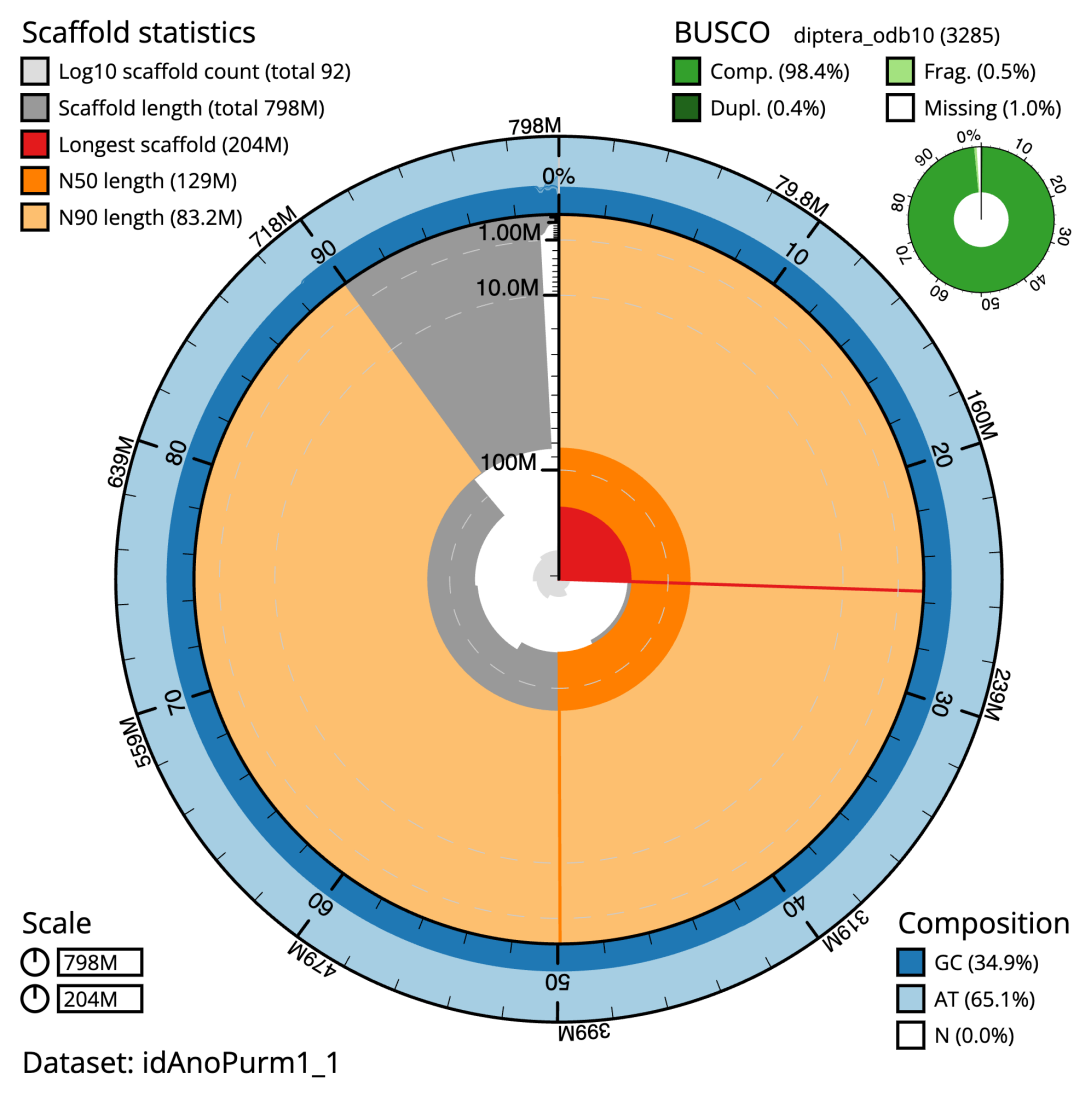
Genome assembly of *Anomoia purmunda*, idAnoPurm1.1: metrics. The BlobToolKit snail plot shows N50 metrics and BUSCO gene completeness. The main plot is divided into 1,000 size-ordered bins around the circumference with each bin representing 0.1% of the 798,274,135 bp assembly. The distribution of scaffold lengths is shown in dark grey with the plot radius scaled to the longest scaffold present in the assembly (203,619,140 bp, shown in red). Orange and pale-orange arcs show the N50 and N90 scaffold lengths (129,230,719 and 83,209,835 bp), respectively. The pale grey spiral shows the cumulative scaffold count on a log scale with white scale lines showing successive orders of magnitude. The blue and pale-blue area around the outside of the plot shows the distribution of GC, AT and N percentages in the same bins as the inner plot. A summary of complete, fragmented, duplicated and missing BUSCO genes in the diptera_odb10 set is shown in the top right. An interactive version of this figure is available at https://blobtoolkit.genomehubs.org/view/idAnoPurm1_1/dataset/idAnoPurm1_1/snail.

**Figure 3.**
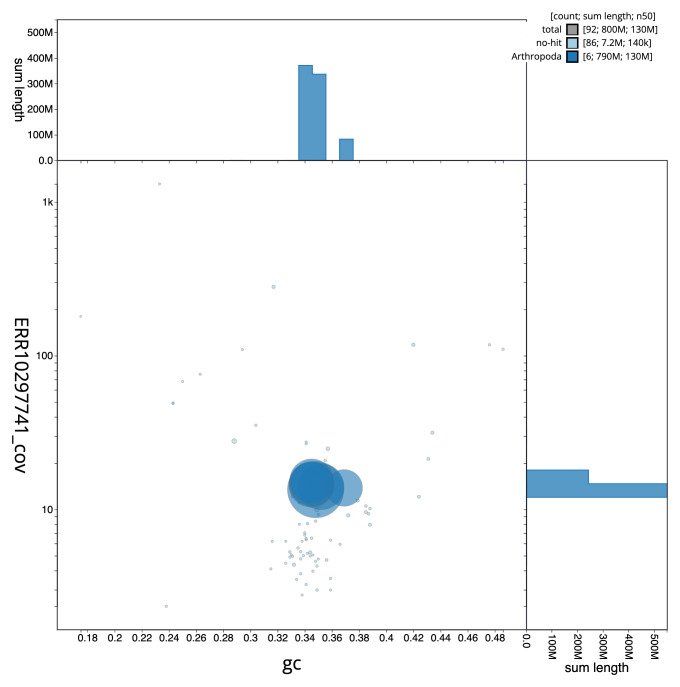
Genome assembly of *Anomoia purmunda*, idAnoPurm1.1: Blob plot of base coverage in ERR10297741 against GC proportion for sequences in the assembly. Sequences are coloured by phylum. Circles are sized in proportion to sequence length. Histograms show the distribution of sequence length sum along each axis. An interactive version of this figure is available at https://blobtoolkit.genomehubs.org/view/idAnoPurm1_1/dataset/idAnoPurm1_1/blob.

**Figure 4.**
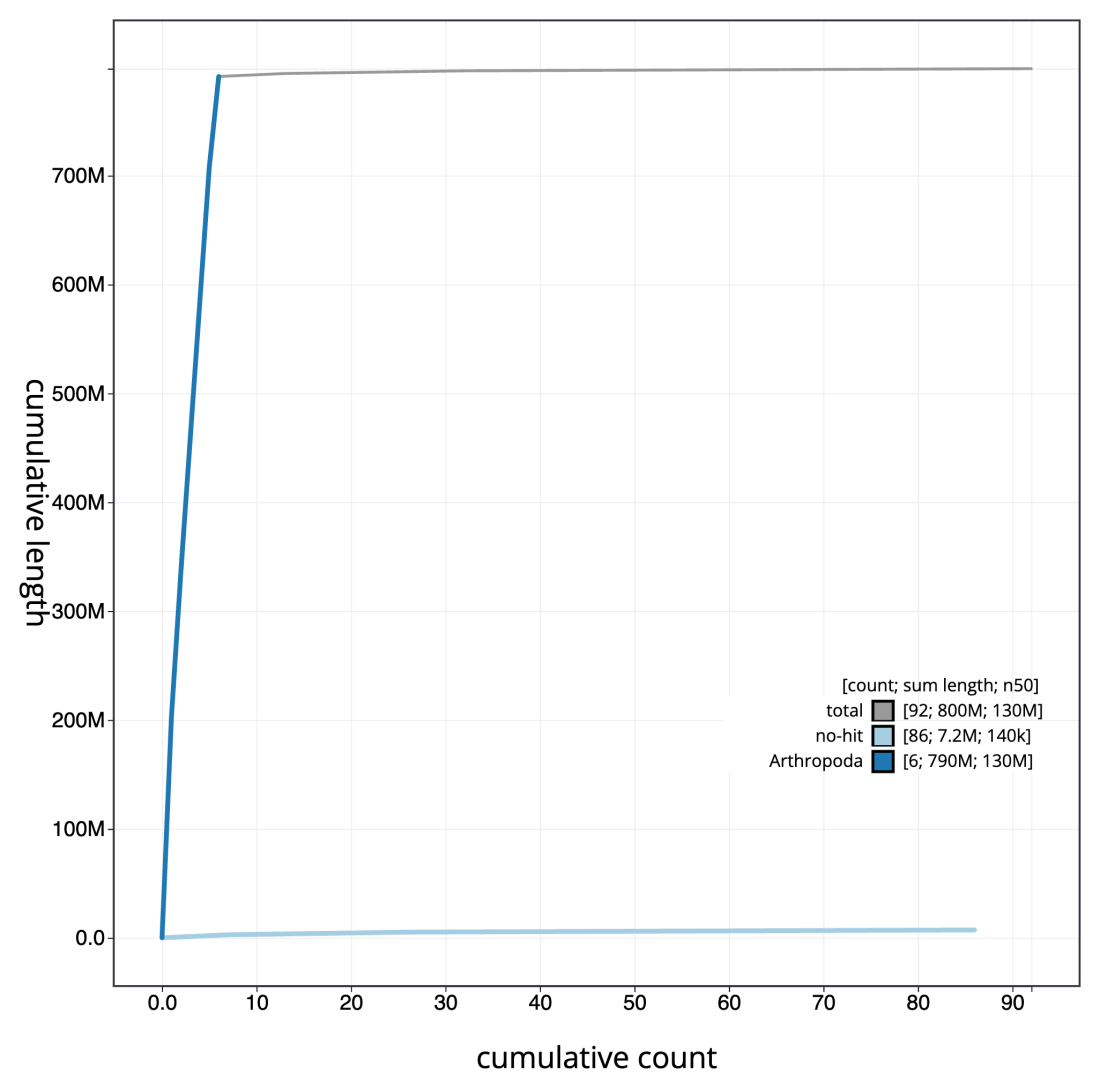
Genome assembly of *Anomoia purmunda* idAnoPurm1.1: BlobToolKit cumulative sequence plot. The grey line shows cumulative length for all sequences. Coloured lines show cumulative lengths of sequences assigned to each phylum using the buscogenes taxrule. An interactive version of this figure is available at https://blobtoolkit.genomehubs.org/view/idAnoPurm1_1/dataset/idAnoPurm1_1/cumulative.

**Figure 5.**
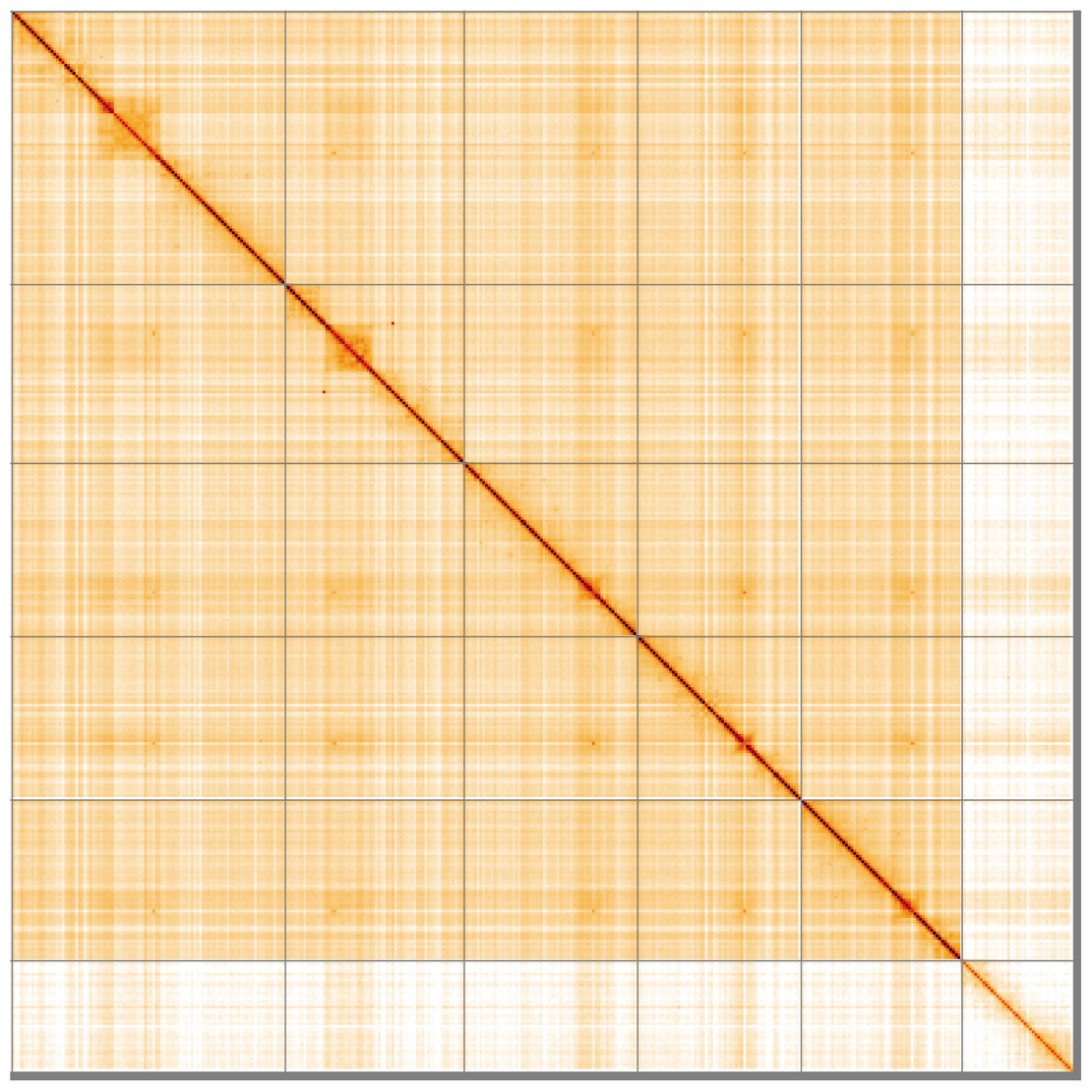
Genome assembly of *Anomoia purmunda*, idAnoPurm1.1: Hi-C contact map of the idAnoPurm1.1 assembly, visualised using HiGlass. Chromosomes are shown in order of size from left to right and top to bottom. An interactive version of this figure may be viewed at https://genome-note-higlass.tol.sanger.ac.uk/l/?d=CMzULYvqQtav0aMtA9XUDQ.

Manual assembly curation corrected 88 missing joins or mis-joins and 2 haplotypic duplications, reducing the assembly length by 0.2% and the scaffold number by 30.83%. The final assembly has a total length of 798.30 Mb in 91 sequence scaffolds, with 443 gaps and a scaffold N50 of 129.2 Mb ( Table 2). The snail plot in Figure 2 provides a summary of the assembly statistics, while the distribution of assembly scaffolds on GC proportion and coverage is shown in Figure 3. The cumulative assembly plot in Figure 4 shows curves for subsets of scaffolds assigned to different phyla. Most (99.56%) of the assembly sequence was assigned to 6 chromosomal-level scaffolds, representing 5 autosomes and the X sex chromosome. Chromosome-scale scaffolds confirmed by the Hi-C data are named in order of size ( Figure 5; Table 3). The X chromosome was assigned based on synteny to *Anastrepha ludens (*GCF_028408465.1). While not fully phased, the assembly deposited is of one haplotype. Contigs corresponding to the second haplotype have also been deposited. The mitochondrial genome was also assembled and can be found as a contig within the multifasta file of the genome submission.

**Table 2.** Genome assembly data for *Anomoia purmunda*, idAnoPurm1.1.

Genome assembly		
Assembly name	idAnoPurm1.1	
Assembly accession	GCA_951828415.1	
*Accession of alternate haplotype*	*GCA_951828255.1*	
Span (Mb)	798.30	
Number of contigs	535	
Contig N50 length (Mb)	7.4	
Number of scaffolds	91	
Scaffold N50 length (Mb)	129.2	
Longest scaffold (Mb)	203.62	
Assembly metrics *		*Benchmark*
Consensus quality (QV)	61.2	*≥ 50*
*k*-mer completeness	100.0%	*≥ 95%*
BUSCO **	C:98.4%[S:98.1%,D:0.4%], F:0.5%,M:1.0%,n:3,285	*C ≥ 95%*
Percentage of assembly mapped to chromosomes	99.56%	*≥ 95%*
Sex chromosomes	X	*localised homologous pairs*
Organelles	Mitochondrial genome: 16.48 kb	*complete single alleles*

* Assembly metric benchmarks are adapted from column VGP-2020 of “Table 1: Proposed standards and metrics for defining genome assembly quality” from Rhie *et al.* (2021). ** BUSCO scores based on the diptera_odb10 BUSCO set using version 5.3.2. C = complete [S = single copy, D = duplicated], F = fragmented, M = missing, n = number of orthologues in comparison. A full set of BUSCO scores is available at https://blobtoolkit.genomehubs.org/view/idAnoPurm1_1/dataset/idAnoPurm1_1/busco.

**Table 3.** Chromosomal pseudomolecules in the genome assembly of *Anomoia purmunda*, idAnoPurm1.

INSDC accession	Name	Length (Mb)	GC%
OX639993.1	1	203.62	35.0
OX639994.1	2	133.27	35.0
OX639995.1	3	129.23	34.5
OX639996.1	4	121.85	34.5
OX639997.1	5	119.9	34.5
OX639998.1	X	83.21	37.0
OX639999.1	MT	0.02	23.5

The estimated Quality Value (QV) of the final assembly is 61.2 with *k*-mer completeness of 100.0%, and the assembly has a BUSCO v5.3.2 completeness of 98.4% (single = 98.1%, duplicated = 0.4%), using the diptera_odb10 reference set ( *n* = 3,285).

Metadata for specimens, BOLD barcode results, spectra estimates, sequencing runs, contaminants and pre-curation assembly statistics are given at https://links.tol.sanger.ac.uk/species/103414.

## Methods

### Sample acquisition

The specimen used for genome sequencing, a female *Anomoia purmunda* (specimen ID Ox000193, ToLID idAnoPurm1) was collected from Lord’s Common, Wytham Woods, Oxfordshire (biological vice-county Berkshire), UK (latitude 51.77, longitude –1.33) on 2019-08-20 by potting. The specimen was collected and identified by Liam Crowley (University of Oxford) and preserved on dry ice.

The specimens used for Hi-C sequencing (specimen ID Ox002317, ToLID idAnoPurm2) and RNA sequencing (specimen ID Ox002677, ToLID idAnoPurm3) were collected from the same location on 2022-07-22 and 2022-08-12 respectively. Specimens idAnoPurm2 and idAnoPurm3 were collected and identified by Liam Crowley and James McCulloch (University of Oxford), and preserved on dry ice.

The initial species identification was verified by an additional DNA barcoding process according to the framework developed by Twyford *et al*. (2024). A small sample was dissected from the specimen and stored in ethanol, while the remaining parts of the specimen were shipped on dry ice to the Wellcome Sanger Institute (WSI). The tissue was lysed, the COI marker region was amplified by PCR, and amplicons were sequenced and compared to the BOLD database, confirming the species identification ( Crowley *et al.*, 2023). Following whole genome sequence generation, the relevant DNA barcode region was also used alongside the initial barcoding data for sample tracking at the WSI ( Twyford *et al.*, 2024). The standard operating procedures for Darwin Tree of Life barcoding have been deposited on protocols.io ( Beasley *et al.*, 2023).

### Nucleic acid extraction

The workflow for high molecular weight (HMW) DNA extraction at the Wellcome Sanger Institute (WSI) includes a sequence of core procedures: sample preparation; sample homogenisation, DNA extraction, fragmentation, and clean-up. In sample preparation, the idAnoPurm1 sample was weighed and dissected on dry ice ( Jay *et al.*, 2023). Tissue from the whole organism was homogenised using a PowerMasher II tissue disruptor ( Denton *et al.*, 2023a).

HMW DNA was extracted using the Manual MagAttract v1 protocol ( Strickland *et al.*, 2023b). DNA was sheared into an average fragment size of 12–20 kb in a Megaruptor 3 system with speed setting 30 ( Todorovic *et al.*, 2023). Sheared DNA was purified by solid-phase reversible immobilisation ( Strickland *et al.*, 2023a): in brief, the method employs AMPure PB beads to eliminate shorter fragments and concentrate the DNA. The concentration of the sheared and purified DNA was assessed using a Nanodrop spectrophotometer and Qubit Fluorometer and Qubit dsDNA High Sensitivity Assay kit. Fragment size distribution was evaluated by running the sample on the FemtoPulse system.

RNA was extracted from whole organism tissue of idAnoPurm3 in the Tree of Life Laboratory at the WSI using the RNA Extraction: Automated MagMax™ *mir*Vana protocol ( do Amaral *et al.*, 2023). The RNA concentration was assessed using a Nanodrop spectrophotometer and a Qubit Fluorometer using the Qubit RNA Broad-Range Assay kit. Analysis of the integrity of the RNA was done using the Agilent RNA 6000 Pico Kit and Eukaryotic Total RNA assay.

Protocols developed by the WSI Tree of Life laboratory are publicly available on protocols.io ( Denton *et al.*, 2023b).

### Sequencing

Pacific Biosciences HiFi circular consensus DNA sequencing libraries were constructed according to the manufacturers’ instructions. Poly(A) RNA-Seq libraries were constructed using the NEB Ultra II RNA Library Prep kit. DNA and RNA sequencing was performed by the Scientific Operations core at the WSI on Pacific Biosciences Sequel II (HiFi) and Illumina NovaSeq 6000 (RNA-Seq) instruments. Hi-C data were also generated from whole organism tissue of idAnoPurm2 using the Arima-HiC v2 kit. The Hi-C sequencing was performed using paired-end sequencing with a read length of 150 bp on the Illumina NovaSeq 6000 instrument.

### Genome assembly, curation and evaluation

***Assembly***

The original assembly of HiFi reads was performed using Hifiasm ( Cheng *et al.*, 2021) with the --primary option. Haplotypic duplications were identified and removed with purge_dups ( Guan *et al.*, 2020). Hi-C reads were further mapped with bwa-mem2 ( Vasimuddin *et al.*, 2019) to the primary contigs, which were further scaffolded using the provided Hi-C data ( Rao *et al.*, 2014) in YaHS ( Zhou *et al.*, 2023) using the --break option. Scaffolded assemblies were evaluated using Gfastats ( Formenti *et al.*, 2022), BUSCO ( Manni *et al.*, 2021) and MERQURY.FK ( Rhie *et al.*, 2020).

The mitochondrial genome was assembled using MitoHiFi ( Uliano-Silva *et al.*, 2023), which runs MitoFinder ( Allio *et al.*, 2020) and uses these annotations to select the final mitochondrial contig and to ensure the general quality of the sequence.

***Assembly curation***

The assembly was decontaminated using the Assembly Screen for Cobionts and Contaminants (ASCC) pipeline (article in preparation). Flat files and maps used in curation were generated in TreeVal ( Pointon *et al.*, 2023). Manual curation was primarily conducted using PretextView ( Harry, 2022), with additional insights provided by JBrowse2 ( Diesh *et al.*, 2023) and HiGlass ( Kerpedjiev *et al.*, 2018). Scaffolds were visually inspected and corrected as described by Howe *et al*. (2021). Any identified contamination, missed joins, and mis-joins were corrected, and duplicate sequences were tagged and removed. The sex chromosome was assigned based on synteny. The entire process is documented at https://gitlab.com/wtsi-grit/rapid-curation (article in preparation).

***Evaluation of the final assembly***

A Hi-C map for the final assembly was produced using bwa-mem2 ( Vasimuddin *et al.*, 2019) in the Cooler file format ( Abdennur & Mirny, 2020). To assess the assembly metrics, the *k*-mer completeness and QV consensus quality values were calculated in Merqury ( Rhie *et al.*, 2020). This work was done using Nextflow ( Di Tommaso *et al.*, 2017) DSL2 pipelines “sanger-tol/readmapping” ( Surana *et al.*, 2023a) and “sanger-tol/genomenote” ( Surana *et al.*, 2023b). The genome was analysed within the BlobToolKit environment ( Challis *et al.*, 2020) and BUSCO scores ( Manni *et al.*, 2021; Simão *et al.*, 2015) were calculated.

Table 4 contains a list of relevant software tool versions and sources.

**Table 4.** Software tools: versions and sources.

Software tool	Version	Source
BlobToolKit	4.2.1	https://github.com/blobtoolkit/blobtoolkit
BUSCO	5.3.2	https://gitlab.com/ezlab/busco
Hifiasm	0.16.1-r375	https://github.com/chhylp123/hifiasm
HiGlass	1.11.6	https://github.com/higlass/higlass
Merqury	MerquryFK	https://github.com/thegenemyers/MERQURY.FK
MitoHiFi	2	https://github.com/marcelauliano/MitoHiFi
PretextView	0.2	https://github.com/wtsi-hpag/PretextView
purge_dups	1.2.3	https://github.com/dfguan/purge_dups
sanger-tol/genomenote	v1.0	https://github.com/sanger-tol/genomenote
sanger-tol/readmapping	1.1.0	https://github.com/sanger-tol/readmapping/tree/1.1.0
YaHS	yahs-1.1.91eebc2	https://github.com/c-zhou/yahs

### Wellcome Sanger Institute – Legal and Governance

The materials that have contributed to this genome note have been supplied by a Darwin Tree of Life Partner. The submission of materials by a Darwin Tree of Life Partner is subject to the **‘Darwin Tree of Life Project Sampling Code of Practice’**, which can be found in full on the Darwin Tree of Life website here. By agreeing with and signing up to the Sampling Code of Practice, the Darwin Tree of Life Partner agrees they will meet the legal and ethical requirements and standards set out within this document in respect of all samples acquired for, and supplied to, the Darwin Tree of Life Project.

Further, the Wellcome Sanger Institute employs a process whereby due diligence is carried out proportionate to the nature of the materials themselves, and the circumstances under which they have been/are to be collected and provided for use. The purpose of this is to address and mitigate any potential legal and/or ethical implications of receipt and use of the materials as part of the research project, and to ensure that in doing so we align with best practice wherever possible. The overarching areas of consideration are:

•   Ethical review of provenance and sourcing of the material

•   Legality of collection, transfer and use (national and international) 

Each transfer of samples is further undertaken according to a Research Collaboration Agreement or Material Transfer Agreement entered into by the Darwin Tree of Life Partner, Genome Research Limited (operating as the Wellcome Sanger Institute), and in some circumstances other Darwin Tree of Life collaborators.

## Data Availability

European Nucleotide Archive: Anomoia purmunda (hawthorn fruit fly). Accession number PRJEB55940; https://identifiers.org/ena.embl/PRJEB55940 ( Wellcome Sanger Institute, 2023). The genome sequence is released openly for reuse. The *Anomoia purmunda* genome sequencing initiative is part of the Darwin Tree of Life (DToL) project. All raw sequence data and the assembly have been deposited in INSDC databases. The genome will be annotated using available RNA-Seq data and presented through the Ensembl pipeline at the European Bioinformatics Institute. Raw data and assembly accession identifiers are reported in Table 1 and Table 2.

## Author information

Members of the University of Oxford and Wytham Woods Genome Acquisition Lab are listed here: https://doi.org/10.5281/zenodo.12157525.

Members of the Darwin Tree of Life Barcoding collective are listed here: https://doi.org/10.5281/zenodo.12158331

Members of the Wellcome Sanger Institute Tree of Life Management, Samples and Laboratory team are listed here: https://doi.org/10.5281/zenodo.12162482.

Members of Wellcome Sanger Institute Scientific Operations: Sequencing Operations are listed here: https://doi.org/10.5281/zenodo.12165051.

Members of the Wellcome Sanger Institute Tree of Life Core Informatics team are listed here: https://doi.org/10.5281/zenodo.12160324.

Members of the Tree of Life Core Informatics collective are listed here: https://doi.org/10.5281/zenodo.5013541.

Members of the Darwin Tree of Life Consortium are listed here: https://doi.org/10.5281/zenodo.4783558.

## References

[ref-1] Abdennur N Mirny LA : Cooler: scalable storage for Hi-C data and other genomically labeled arrays. *Bioinformatics.* 2020;36(1):311–316. 10.1093/bioinformatics/btz540 31290943 PMC8205516

[ref-2] Allio R Schomaker-Bastos A Romiguier J : MitoFinder: efficient automated large-scale extraction of mitogenomic data in target enrichment phylogenomics. *Mol Ecol Resour.* 2020;20(4):892–905. 10.1111/1755-0998.13160 32243090 PMC7497042

[ref-3] Beasley J Uhl R Forrest LL : DNA barcoding SOPs for the Darwin Tree of Life project. *protocols.io.* 2023; [Accessed 25 June 2024]. 10.17504/protocols.io.261ged91jv47/v1

[ref-4] Challis R Richards E Rajan J : BlobToolKit – interactive quality assessment of genome assemblies. *G3 (Bethesda).* 2020;10(4):1361–1374. 10.1534/g3.119.400908 32071071 PMC7144090

[ref-5] Cheng H Concepcion GT Feng X : Haplotype-resolved *de novo* assembly using phased assembly graphs with hifiasm. *Nat Methods.* 2021;18(2):170–175. 10.1038/s41592-020-01056-5 33526886 PMC7961889

[ref-6] Crowley L Allen H Barnes I : A sampling strategy for genome sequencing the British terrestrial arthropod fauna [version 1; peer review: 2 approved]. *Wellcome Open Res.* 2023;8:123. 10.12688/wellcomeopenres.18925.1 37408610 PMC10318377

[ref-7] Denton A Oatley G Cornwell C : Sanger Tree of Life sample homogenisation: PowerMash. *protocols.io.* 2023a. 10.17504/protocols.io.5qpvo3r19v4o/v1

[ref-8] Denton A Yatsenko H Jay J : Sanger Tree of Life wet laboratory protocol collection V.1. *protocols.io.* 2023b. 10.17504/protocols.io.8epv5xxy6g1b/v1

[ref-9] Di Tommaso P Chatzou M Floden EW : Nextflow enables reproducible computational workflows. *Nat Biotechnol.* 2017;35(4):316–319. 10.1038/nbt.3820 28398311

[ref-10] Diesh C Stevens GJ Xie P : JBrowse 2: a modular genome browser with views of synteny and structural variation. *Genome Biol.* 2023;24(1):74. 10.1186/s13059-023-02914-z 37069644 PMC10108523

[ref-11] do Amaral RJV Bates A Denton A : Sanger Tree of Life RNA extraction: automated MagMax ^TM^ mirVana. *protocols.io.* 2023. 10.17504/protocols.io.6qpvr36n3vmk/v1

[ref-12] Formenti G Abueg L Brajuka A : Gfastats: conversion, evaluation and manipulation of genome sequences using assembly graphs. *Bioinformatics.* 2022;38(17):4214–4216. 10.1093/bioinformatics/btac460 35799367 PMC9438950

[ref-13] GBIF.org: Occurrence download.2024; [Accessed 23 June 2024]. 10.15468/DL.KTPC7X

[ref-14] Greene E Orsak LJ Whitman DW : A tephritid fly mimics the territorial displays of its jumping spider predators. *Science.* 1987;236(4799):310–312. 10.1126/science.236.4799.310 17755555

[ref-15] Guan D McCarthy SA Wood J : Identifying and removing haplotypic duplication in primary genome assemblies. *Bioinformatics.* 2020;36(9):2896–2898. 10.1093/bioinformatics/btaa025 31971576 PMC7203741

[ref-16] Harry E : PretextView (Paired REad TEXTure Viewer): a desktop application for viewing pretext contact maps.2022; [Accessed 19 October 2022]. Reference Source

[ref-17] Hoffmeister T : Factors determining the structure and diversity of parasitoid complexes in tephritid fruit flies. *Oecologia.* 1992;89(2):288–297. 10.1007/BF00317230 28312885

[ref-18] Howe K Chow W Collins J : Significantly improving the quality of genome assemblies through curation. *GigaScience.* 2021;10(1): giaa153. 10.1093/gigascience/giaa153 33420778 PMC7794651

[ref-19] Jay J Yatsenko H Narváez-Gómez JP : Sanger Tree of Life sample preparation: triage and dissection. *protocols.io.* 2023. 10.17504/protocols.io.x54v9prmqg3e/v1

[ref-20] Kandybina MN : Contribution to the study of the Tephritidae (Dipt.) of the Mongolian People’s Republic. *Ent Obozr.* 1972;51:909–918.

[ref-21] Kandybina MN : The larvae of fruit-flies (Diptera, Tephritidae). [In Russian: English summary in Rev. appl. Ent. Series A 66: 2961.]. *Opred Faune SSSR.* 1977;114:1–212.

[ref-22] Kerpedjiev P Abdennur N Lekschas F : HiGlass: web-based visual exploration and analysis of genome interaction maps. *Genome Biol.* 2018;19(1): 125. 10.1186/s13059-018-1486-1 30143029 PMC6109259

[ref-23] Korneyev VA Korneyev SV : First records of the trypetine mining flies (Diptera: Tephritidae: Trypetini) from Ukraine. *Ukr Entomofaunistyka.* 2015;6(3):45–47. Reference Source

[ref-24] Manni M Berkeley MR Seppey M : BUSCO update: novel and streamlined workflows along with broader and deeper phylogenetic coverage for scoring of eukaryotic, prokaryotic, and viral genomes. *Mol Biol Evol.* 2021;38(10):4647–4654. 10.1093/molbev/msab199 34320186 PMC8476166

[ref-25] NBN Trust Partnership: *Anomoia purmunda* map. NBN atlas.2024; [Accessed 23 June 2024]. Reference Source

[ref-26] Pointon DL Eagles W Sims Y : sanger-tol/treeval v1.0.0 – Ancient Atlantis.2023. 10.5281/zenodo.10047654

[ref-27] Rao SSP Huntley MH Durand NC : A 3D map of the human genome at kilobase resolution reveals principles of chromatin looping. *Cell.* 2014;159(7):1665–1680. 10.1016/j.cell.2014.11.021 25497547 PMC5635824

[ref-28] Rhie A McCarthy SA Fedrigo O : Towards complete and error-free genome assemblies of all vertebrate species. *Nature.* 2021;592(7856):737–746. 10.1038/s41586-021-03451-0 33911273 PMC8081667

[ref-29] Rhie A Walenz BP Koren S : Merqury: reference-free quality, completeness, and phasing assessment for genome assemblies. *Genome Biol.* 2020;21(1): 245. 10.1186/s13059-020-02134-9 32928274 PMC7488777

[ref-30] Simão FA Waterhouse RM Ioannidis P : BUSCO: assessing genome assembly and annotation completeness with single-copy orthologs. *Bioinformatics.* 2015;31(19):3210–3212. 10.1093/bioinformatics/btv351 26059717

[ref-31] Smith K : An aggregation of *Anomoia purmunda* (Harris) (Dipt., Tephritidae). *Entomol Mon Mag.* 1995;128:74.

[ref-32] Stalažs A : New records of tephritoidea (Diptera: Brachycera) for the fauna of Latvia. *Zool Ecol.* 2014a;24(4):347–351. 10.1080/21658005.2014.939883

[ref-33] Stalažs A : New records of some dipterans (Diptera: Cecidomyidae, Tephritidae) in north-eastern Lithuania. *Zool Ecol.* 2014b;24(1):55–57. 10.1080/21658005.2014.883831

[ref-34] Strickland M Cornwell C Howard C : Sanger Tree of Life fragmented DNA clean up: manual SPRI. *protocols.io.* 2023a. 10.17504/protocols.io.kxygx3y1dg8j/v1

[ref-35] Strickland M Moll R Cornwell C : Sanger Tree of Life HMW DNA extraction: manual MagAttract. *protocols.io.* 2023b. 10.17504/protocols.io.6qpvr33novmk/v1

[ref-36] Surana P Muffato M Qi G : sanger-tol/readmapping: sanger-tol/readmapping v1.1.0 - Hebridean Black (1.1.0). *Zenodo.* 2023a. 10.5281/zenodo.7755669

[ref-37] Surana P Muffato M Sadasivan Baby C : sanger-tol/genomenote (v1.0.dev). *Zenodo.* 2023b. 10.5281/zenodo.6785935

[ref-38] Tartanus M Malusá E Furmańczyk EM : Monitoring fruit fly populations in cherry, Japanese rose and sea buckthorn in organic orchards in Poland.In: *Understanding pests and their control agents as the basis for integrated plant protection: Proceedings of the VIII Congress on Plant Protection*.2021.

[ref-39] Teodoru A Chiriloaie A Chireceanu C : The hawthorn fruit fly, *Anomoia purmunda* Harris-A less known species in Romania. *Romanian J Plant Prot.* 2015;8:47–53. Reference Source

[ref-40] Thompson I Blunt G : Invertebrate communities of old traditional orchards in south Shropshire (VC40). Field Studies Council,2018. Reference Source

[ref-41] Todorovic M Sampaio F Howard C : Sanger Tree of Life HMW DNA fragmentation: diagenode Megaruptor ^®^3 for PacBio HiFi. *protocols.io.* 2023. 10.17504/protocols.io.8epv5x2zjg1b/v1

[ref-42] Tuba K : Data about presence of tree fruitflies ( *Anomoia permunda*, *Carpomya schineri* and *Campiglossa grandinata*) in county Vas. *Növényvédelem.* 2009;45:491–495.

[ref-43] Twyford AD Beasley J Barnes I : A DNA barcoding framework for taxonomic verification in the Darwin Tree of Life Project [version 1; peer review: awaiting peer review]. *Wellcome Open Res.* 2024;9:339. 10.12688/wellcomeopenres.21143.1 PMC11462125 39386966

[ref-44] Uliano-Silva M Ferreira JGRN Krasheninnikova K : MitoHiFi: a python pipeline for mitochondrial genome assembly from PacBio high fidelity reads. *BMC Bioinformatics.* 2023;24(1): 288. 10.1186/s12859-023-05385-y 37464285 PMC10354987

[ref-45] Vasimuddin M Misra S Li H : Efficient architecture-aware acceleration of BWA-MEM for multicore systems.In: *2019 IEEE International Parallel and Distributed Processing Symposium (IPDPS).*IEEE,2019;314–324. 10.1109/IPDPS.2019.00041

[ref-46] Wellcome Sanger Institute: The genome sequence of the hawthorn fruit fly, *Anomoia purmunda* (Harris, 1780). European Nucleotide Archive.[dataset], accession number PRJEB55940,2023.

[ref-47] White IM : Tephritid flies: diptera: tephritidae, 1.In: *Handbooks for the identification of British insects*. London: Royal Entomological Society of London,1988.

[ref-48] Zhou C McCarthy SA Durbin R : YaHS: yet another Hi-C scaffolding tool. *Bioinformatics.* 2023;39(1): btac808. 10.1093/bioinformatics/btac808 36525368 PMC9848053

