# Supplementary material for: The genome sequence of the Hawthorn Fruit Fly, Anomoia purmunda (Harris, 1780)
Source: Wellcome Open Res. 2024 Aug 12;9:466. [Version 1] doi: 10.12688/wellcomeopenres.22849.1 (PMC11447438; doi:10.12688/wellcomeopenres.22849.1)
Supplement: Peer review history

# Reviewer report: Reviewer response for version 1

**DOI:** 10.21956/wellcomeopenres.25157.r98537

**Published:** 2024-10-02

**Authors:** Stacey L. Hanlon

**Affiliations:** 1 University of Connecticut, Storrs, Connecticut, USA

Overall, this is a solid report of the genome sequence for *Anomoia purmunda*.  I am impressed with the amount of genomic data that was able to be extracted from three individual specimens. 

 I concur with Dr. Kareem's expert review that very clearly an thoroughly highlighted a few areas where the manuscript could be improved.  It is highly recommended that a few additional details regarding pre-assembly QC be included, and an general analysis of the findings would be appreciated.  For example, the ENA project has the raw genomic data corresponding to *Anomoia purmunda*, but it also has the sequence of the *Wolbachia* that was presumably living inside its cells.  There was no mention of this in the manuscript; I'm not sure if it was a purposeful omission, but it would be interesting to note that it was found in this sample.  Also, the interactive figures were helpful for helping me understand the data on a deeper level.

Are sufficient details of methods and materials provided to allow replication by others?

Partly

Is the rationale for creating the dataset(s) clearly described?

Yes

Are the datasets clearly presented in a useable and accessible format?

Yes

Are the protocols appropriate and is the work technically sound?

Yes

Reviewer Expertise:

Drosophila genetics

I confirm that I have read this submission and believe that I have an appropriate level of expertise to confirm that it is of an acceptable scientific standard.

# Reviewer report: Reviewer response for version 1

**DOI:** 10.21956/wellcomeopenres.25157.r98539

**Published:** 2024-09-23

**Authors:** Ali A. Kareem

**Affiliations:** 1 University of Kerbala, Karbala, Karbala Governorate, Iraq

The described procedure for genome sequencing of * Anomoia purmunda* covers several essential steps. It is intensive work and great results.  However, there are a few potential points that could be expanded or clarified:

**1. Sequencing Platform Details:** The procedure mentions using Pacific Biosciences HiFi long reads and Hi-C data but omits important technical parameters like read length, sequencing depth justification, and why these platforms were selected over others (e.g., Oxford Nanopore).

**2. Quality Control Metrics:** It doesn't specify pre-assembly quality control steps like read trimming, filtering, or error correction applied to raw sequencing data. These steps are crucial for ensuring high-quality assemblies.

**3. Assembly Tools:** The description lacks details on the software and algorithms used for genome assembly and scaffolding (e.g., tools like Canu, Flye, or Hi-C scaffolding software like Juicer). Additionally, the decision-making process for resolving mis-joins and duplications is unclear.

**4. Phasing Explanation:** The assembly mentions being "not fully phased," but further clarification on how phasing was handled and whether it affects the interpretation of heterozygosity or structural variants could be helpful.

**5. Annotation of Functional Elements:** There's no mention of how gene annotation, repeat identification, or structural variant calling was performed. Annotation pipelines (e.g., MAKER or Augustus) or comparative genomics tools (BLAST or InterProScan) should be noted.

**6. Validation of Chromosomes:** While synteny with *Anastrepha ludens* is referenced, the specific methods for synteny analysis and further validation of chromosome assembly (e.g., use of additional references or manual curation) are not explained.

**7. Mitochondrial Genome Assembly:** The mitochondrial genome assembly is mentioned but lacks details on specific assembly parameters and how it was verified (e.g., by comparison to known mitochondrial sequences or structures).

**8. Post-Assembly Analysis:** There’s no mention of additional post-assembly analysis, such as variant calling, structural rearrangements, or comparative genomics, that could further improve the utility of the genome data.

 9. It will be great to identify the symbionts of *Anomoia purmunda *molecularly to know the species of endosymbionts in future work.

Are sufficient details of methods and materials provided to allow replication by others?

Yes

Is the rationale for creating the dataset(s) clearly described?

Yes

Are the datasets clearly presented in a useable and accessible format?

Yes

Are the protocols appropriate and is the work technically sound?

Yes

Reviewer Expertise:

Genetic Diversity and Molecular Taxonomy of Insects

I confirm that I have read this submission and believe that I have an appropriate level of expertise to confirm that it is of an acceptable scientific standard.
